# Drinking-water efficiency, cost of illness, and peri-urban society: An economic household analysis

**DOI:** 10.1371/journal.pone.0257509

**Published:** 2021-09-29

**Authors:** Ghaffar Ali, Muhammad Khalid Bashir, Sawaid Abbas, Mehwish Murtaza

**Affiliations:** 1 College of Management, Shenzhen University, Shenzhen, Guangdong, China; 2 Institute of Agricultural and Resource Economics, University of Agriculture Faisalabad, Faisalabad, Pakistan; 3 Department of Land Surveying and Geo-informatics, The Hong Kong Polytechnic University, Hong Kong, SAR China; International Centre for Integrated Mountain Development (ICIMOD), Kathmandu, Nepal, NEPAL

## Abstract

This study aims to measure the efficacy of drinking water in terms of the economic impacts and risk of illness involved in using perilous water sources. Socio-economic factors were also considered. A multidisciplinary approach was employed to analyze the data, including the cost of illness (COI), regression technique, and irrigation water efficiency methods. The primary data set consisted of 210 peri-urban and urban households. It was found that the average cost of illness was higher in peri-urban ($10.79 USD) areas, while willingness to pay for quality water was higher in urban residents. Social status, income, and family size was positively associated with the cost of illness, while education, the source of drinking water (ground water and others), and awareness about safe drinking were negatively associated with the cost of illness. Furthermore, urban residents were more efficient in terms of conveyance and water use. This is one of the first studies to apply irrigation water efficiency methods to measure drinking water efficiency. The results are timely and important with both practical and social implications, including guiding policy framework. It is suggested that family planning programs be made more effective to control family size. The filtration plants to enhance drinking water quality be installed in the central places of each town/division/union council. A public-private partnership could work to provide affordable quality drinking water.

## 1. Introduction

Access to adequate and safe drinking water is the basic necessity of humans. Safe drinking water plays an important role in reducing health care costs [1], and globally, it helps control the incidence of waterborne diseases [2]. More than 850 million people throughout the world have limited or no access to clean drinking water [3], which often causes outbreaks of many dangerous water-borne diseases, such as diarrhea, typhoid, and hepatitis [4]. It is well established that a majority of these people live in Africa and Asia [5]. Pakistan is the sixth most populous country in the world, with a population of more than 180 million that is expected to surpass 240 million by 2030 [6]. The demand for safe drinking water and water used for domestic, agricultural and industrial purposes is expected to rise significantly. Only a few decades ago, Pakistan was a water rich country, though it is now among the 17 countries with the highest levels of water scarcity, with an annual per capita fresh water availability of less than 1000 cubic meters. The drinking water treatment facilities and infrastructure has been seriously neglected in Pakistan. Approximately 60% of Pakistan’s population does not have access to safe drinking water [7]. The access issue is more severe in rural and peri-urban areas, where water scarcity affects 90% of the population.

In Pakistan, groundwater is the major source of drinking water for more than 60% of the population, and it is retrieved using hand and motorized pumps. More than 70% of the rural and peri-urban households use groundwater for drinking purposes. Due to heavy pumping and low recharge rate, availability of ground water is expected to further decline in the future [8, 9]. Groundwater quality is often questioned due to rapid population growth, unplanned urbanization [10], contamination of industrial wastewater, mixing or leaching effects associated with domestic sewage, and, other anthropogenic activities [11]. The low quality of drinking water is causing the spread of water-borne diseases, which is the major reason for the increase in unsustainable drinking practices [5, 12, 13] that lead to an increase in the cost of living (i.e., increased water and health expenditures) [11].

**Fig 1:**
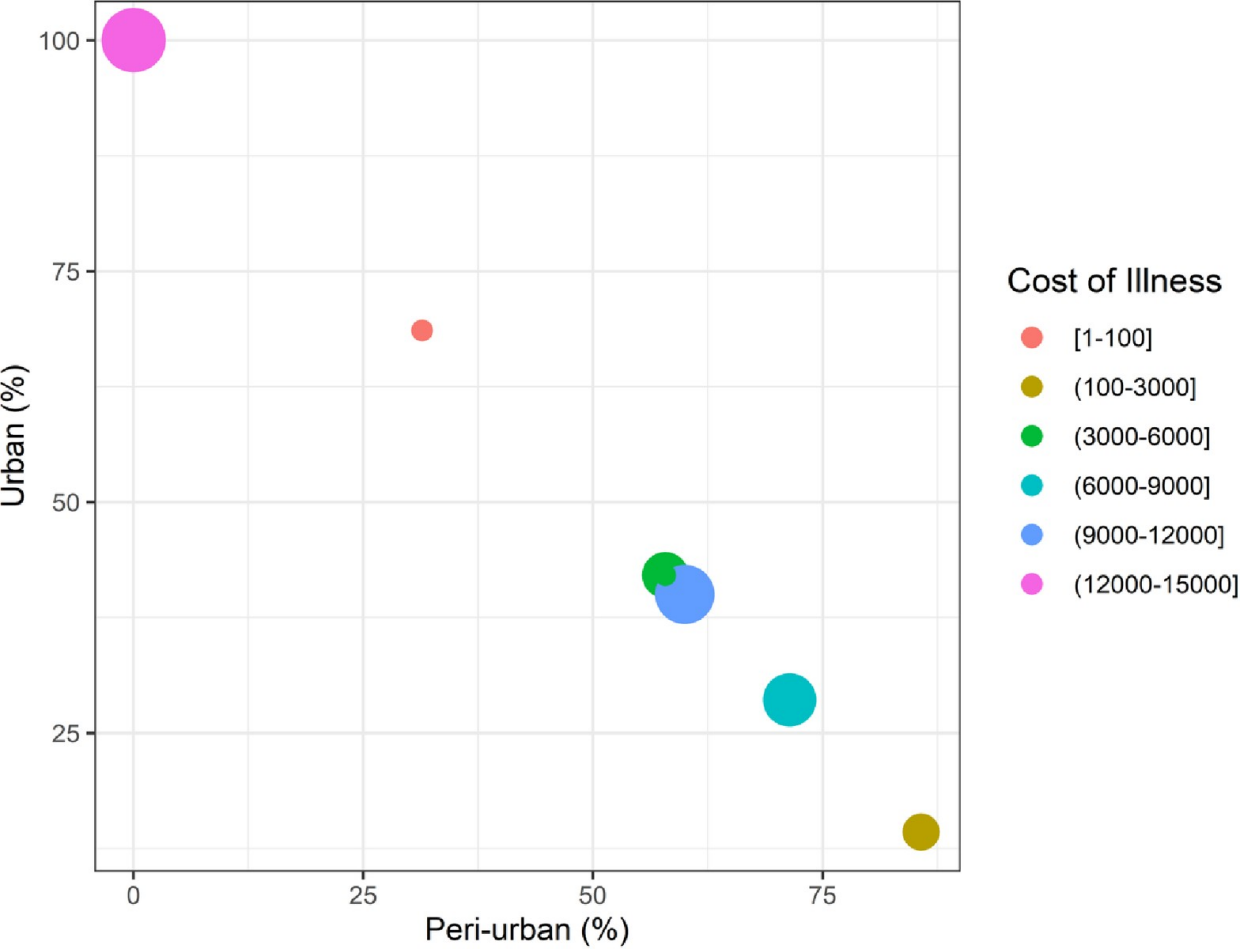
Distribution of cost of illness and social cost of households among urban and peri-urban communities.

Faisalabad is the third largest city of Pakistan. It is famous for its textile industry. The chemicals used in these industrial operations not only pollutes the surface environment, but they also deteriorate the quality of groundwater when discharged into the sewage waste streams. Given the absence of efficient drainage systems and adequate treatment facilities, industrial wastewater is discharged directly into the domestic sewage system without any treatment. Most of this sewage water is used to irrigate agricultural crops in peri-urban areas, which is one of the major causes of dangerous chemical residue accumulation on vegetables and other crops, which has a direct negative impact on human health. The leaching effect (mostly due to damaged sewage pipes) result in the mixing of these water contaminants directly with the groundwater, which significantly decreases groundwater quality [14]. Fig 1 depicts the actual conditions captured from one of study areas’ locality. The Water and Sanitation Agency (WASA) is responsible for planning, designing, and constructing water supply, sewage, and drainage infrastructure in Pakistan. It is non-functional in many peri-urban areas and wastewater infrastructure is also substandard. Moreover, the devastation inflicted on water bodies due to waste affects the odor and taste of the groundwater, which is the prime source of drinking water in peri-urban and most urban areas of Faisalabad. These influential factors degrade water quality and cause serious health related threats to the population of Faisalabad [15].

The researchers have focused on willingness to pay [16, 17] (see Vásquez et al., 2009 in Mexico; Khan et al., 2010 in Pakistan; [18] in China; [16] in Bangladesh; [19] in Vietnam; and [20] in Uganda); economic analysis, such as cost of illness (see Cameron et al., 2011 [21] in South Africa; [22] in Pakistan; and [23] in India); water pricing (see Hunter et al., 2009 [24] for developing countries; [18] in China; and [25] in China); and the health effects of unsafe drinking water (see Shygonskyj and Shygonska, 2016 [26] in Ukraine; [27] in Bangladesh; and [28] in Italy). This approach, however, does not capture the complete impact of the consumption of unsafe drinking water because this method ignores (a) individual preference for avoiding pain and suffering as well as (b) individuals’ savings, anxiety, and risk attitude [29–33]. Studies covering the cost of illness and the willingness to pay help policymakers improve policy structure. A third aspect, an efficiency analysis of existing systems and customs, may provide additional information for the policymaking process. To the best of our knowledge, drinking water efficiency analysis is rarely conducted, especially in the case of Faisalabad.

The efficiency of the drinking water system is linked with its intended use without losses that make humans efficient. The efficiency analyses give insights about consumer behavior towards the drinking water and its relationship with labor efficiency. This study aims to improve drinking water policy by filling this research gap.

The objectives of the study are: to estimate the economic impact (cost of illness) of consuming unsafe drinking water; identifying the factors affecting the cost of illness; and measure the efficiency of drinking water. To the best of our knowledge, drinking water efficiency analysis is rarely conducted in the case of Faisalabad. The efficiency of the drinking water system is linked with its method of use without wasting it. The efficiency analyses give insights about consumer behavior towards the drinking water and its relationship with labor efficiency. This study aims to fill this research gap. The objectives of the study are to measure the efficiency of drinking water; estimate the economic impact (cost of illness) of consuming unsafe drinking water; and identifying the factors affecting the cost of illness. Moreover, to identify policy implications to improve the access to safe drinking water in urban and peri-urban areas of the developing country (Pakistan).

## 2. Materials and methods

### 2.1. Data collection

Primary data were collected using a stratified sampling technique from three peri-urban and urban areas, each from Faisalabad city. "No human or animal was harmed during this research study. Verbal consent was taken prior to filling the form, and the data was analyzed anonymously. Since this was a non-experimental, voluntary survey, no ethical approval was required. Additionally, an informed consent form embedded in the survey informed participants about the introduction (voluntary nature of the survey and background), purpose, procedure, risks, benefits, and privacy protection, and the data was analysed anonymously, i.e. no personal identifiers will be presented in the publication. Before beginning the survey, potential participants were made sure that they have read the consent information and agree to participate." From each area, 35 households were randomly selected which formulated a total sample size of 210 households i.e. (35*3)*2 (105 households each from urban and peri-urban areas). A well-structured and pre-tested questionnaire was used to collect the required information from the households.

### 2.2. Economic impact analysis

The cost of illness (COI) method was applied to measure the economic impact of consuming unsafe drinking water. COI estimates cost associated with various water-borne diseases and are regarded as one of the most effective evaluation methods to assess the health impacts of unsafe drinking water [34].

The COI, for this study, was calculated focusing on three dimensions of the expenditures on various illnesses i.e., direct costs, indirect costs, and social costs. The direct costs (DC) include doctor visits (fee), medicine costs, and hospital expenditures. The indirect costs (IC) cover the transportation costs. Moreover, loss of work was considered as a proxy for social costs (SC). Mathematically it can be written as:

COIi=DCi+ICi+SCi (1)

Furthermore, the information on the willingness to pay for quality / safe drinking water was also assessed. The respondents were asked whether they were willing to pay for quality / safe drinking water. In the case of a positive reply, they were further asked as to how much they would like to spend?

### 2.3. Econometric analysis

Following Kim and Park, 2015, Regression analysis (linear) was applied to identify the factors affecting the cost of illness:

$$COI=\beta_{0}+\beta_{1}SS+\beta_{2}AAA+\beta_{3}IOI+\beta_{4}Edu+\beta_{5}HHS+\beta_{6}MI+\beta_{7}SDW+\beta_{8}Aw+\beta_{9}Suf+\beta_{10}Satis+\beta_{11}Avai+\mu$$ (2)

where:

COI = the economic cost due to illnesses caused by the consumption of unsafe drinking water (Pakistan Rupees (PKR)),

SS = Social status (resident of the peri-urban or urban area, dummy variable i.e. 1 for urban and 0 otherwise)

AAA = Average affected Age of household heads (years)

IOI = Incidence of illness (0 for none, 1 for female,2 for male and 3 for both)

Edu = Education (years)

HHS = Household size (numbers)

MI = Monthly income (PKR)

SDW = Source of drinking water (dummy i.e. 1 for groundwater and 0 otherwise)

Aw = Awareness about the importance of safe drinking water (dummy i.e. 1 for aware off and 0 otherwise)

Suff = Household heads’ perception about the incidence of illness due to low quality of the water (dummy i.e. 1 for happened and 0 otherwise)

Satis = Household heads’ satisfaction about the quality of their water (dummy i.e. 1 for satisfied and 0 otherwise)

Avai = Easy availability of quality drinking water (Dummy = = > 1 for easily available and 0 otherwise)

β_0_ = Intercept

β_1–11_ = Coefficients of the variables

Scatter diagrams suggested the linear regression. Furthermore, different forms of the regression were also analyzed but, the results of the linear model were the best. We used Microsoft Excel and SPSS for data analyses.

### 2.4. Efficiency analysis

The efficiency of the drinking water system is linked to its method of use without wasting it. Water efficiency is usually regarded as reducing the water wastage by measuring the difference between the required amount of water for a particular purpose, for example drinking, and the amount of water consumed [33]. This paper adopts irrigation water efficiency measurement methods (see for example Rogars et al., 1997) [35–37] to measure drinking water efficiency. The following methods were adopted with appropriate changes for human water consumption requirements:

#### 2.4.1 Conveyance efficiency

The conveyance efficiency is indicated by the percentage of daily water consumption to its availability:

$$E_{c}=100\;(W_{ci}/W_{ai})$$ (3)

where, E_c_ is conveyance efficiency, W_ci_ is daily water consumption (liters) of i^th^ household and W_ai_ is the total drinking water availability (liters) to the i^th^ household.

#### 2.4.2 Application efficiency

The application efficiency is indicated by the percentage of fulfilling the daily drinking water requirements of a household:

$$E_{a}=100\;(W_{ci}/W_{ri})$$ (4)

where, E_a_ is application efficiency and W_ri_ is the minimum daily water requirement (liters).

A higher percentage is desirable in both the urban and peri-urban areas.

#### 2.4.3 Utilization efficiency

Water use efficiency is usually measured through the cost of extraction or delivery of water and its losses. However, in our case, the proxy of cost of illness was used to represent the outcome of the water consumed (in liters). A lower ratio will show better efficiency of the water consumed in terms of low health costs (which are associated with poor quality of drinking water) that implies an efficient labor output. The water utilization efficiency is measured in terms of the ratio of cost of illness (per day) and daily water consumption:

$$E_{u}=100(COI_{i}/W_{ci})$$ (5)

A lower percentage is desirable in case of utilization efficiency.

## 3. Results and discussion

### 3.1. Economic impact analysis

Households have to bear large expenditures due to the incidence of waterborne diseases. Treatment of drinking water may reduce the cost of illness, due to improved water quality [29–31]. The results of this study show that on average a household had to pay the Pakistani Rupee (PKR) 1,725 (10.79 US$) in peri-urban areas and PKR 1,094 (6.84 US$) in urban areas for the treatment of waterborne diseases (Exchange rate as of 24/07/2021: 1 PKR = 0.0080 US$). The cost ranged from as low as zero to over PKR 11,100 (69.42 US$) in peri-urban areas and about PKR 14,900 (93.19 US$) in urban areas (Table 1). The minimum cost of illness was recorded because some households reported there was no drinking water–associated illness in the household. Furthermore, the difference in the maximum and mean values of urban and peri-urban areas was due to the frequency distribution of the costs. There was only one household in the urban areas whose cost was as high as PKR 14,900, while the majority of the observations were concentrated around PKR 1000. For the peri-urban areas, the majority of the observations were around PKR 1500.

**Table 1:** Cost of illness due to water-borne diseases (PKR).

Stratum	Maximum	Mean	Minimum
Peri-urban	11,100	1724.59 (2349.53)	0
Urban	14,900	1093.89 (2697.37)	0

Figures in parentheses are the standard deviations.

Such costs can threaten both micro- and macro-economies [30]. Previously, Malik et al. [35] found that the cost of illness was about PKR 700 (4.38 US$) in rural areas of Lahore, Pakistan.

Households were asked about their willingness to pay for quality/safe drinking water in terms of an additional cost to their existing water costs. In peri-urban areas 52% of the households were willing to pay for quality/safe drinking water, in contrast to 89% of the urban residents. This implies that the urban residents were more conscious about the quality of drinking water or had better knowledge about waterborne diseases. The frequency distribution for different amounts is presented in Table 2.

**Table 2:** Willingness to pay for quality / safe drinking water.

Willing to pay	Peri-urban	Urban	Total
0	50 (48)	12 (11)	62 (29)
PKR100–150 (0.95–1.43 US$)	12 (11)	0 (0)	12 (6)
PKR 151–300 (1.44–2.86 US$)	38 (36)	31 (30)	69 (33)
PKR 301–400 (2.87–3.82 US$)	4 (4)	15 (14)	19 (9)
PKR 401–500 (3.83–4.77 US$)	1 (1)	26 (25)	27 (13)
PKR 501–600 (4.78–5.73 US$)	0 (0)	13 (12)	13 (6)
PKR 601- up to 1000 (5.74–9.55 US$)	0 (0)	8 (8)	8 (4)
**Total**	**105 (100)**	**105 (100)**	**210 (100)**

Figures in parentheses are the percentages.

### 3.2. Econometric analysis

Following the second objective and research question, the focus is on identification of factors affecting the cost of illness. The results of the regression model are presented in Table 3. The value of R^2^ was 0.642, which indicates that about 64% of the total change in cost of illness was explained by the independent variables. Out of eleven variables, seven were statistically significant. Social status, average affected age, household size, monthly income [36], and perceptions about the incidence of illness due to low-quality water positively impacted the cost of illness, while the education level of the household head had a negative impact.

**Table 3:** Identification of factors affecting COI.

Variables	Β
(Constant)	-1830.5 (486.1) ***
Social status (SS)	914.7 (434.9) **
Average affected Age (AAA)	86.2 (14.7) ***
Incidence of Illness (IOI)	1497.6 (921.8) *
Education (Edu)	-125.5 (105.1) ^NS^
Household size (HHS)	103.2 (41.9) **
Monthly income (MI)	407.5 (147.1) ***
Source of drinking water (SDW)	-94.5 (90.7) ^NS^
Awareness about the importance of safe drinking water (Aw)	-29.8 (353.0) ^NS^
Household heads’ perception of the incidence of illness due to low-quality drinking water (Suff)	1480.8 (566.9) **
Household heads’ satisfaction about the quality of water (Satis)	-985.1 (296.8) ***
Easy availability of quality drinking water (Avail)	-1068.3 (355.6) ***

R^2^ = 0.642 | Figures in parentheses are the standard deviations

*** = significant at less than 1%

** = significant at less than 5%

* = significant at less than 10%

^NS^ = non-significant.

#### 3.2.1. Social status of households (SS)

The cost of illness increased by PKR 915 (8.74 US$) if urban residency increased; this also refers to the value of the coefficient. Cost of illness was substantially related to the social status of peri-urban and urban residents. Usually, the water quality in urban areas is deteriorated due to heavy industrial activity which may be related with an increased cost of illness. Furthermore, the urban centers are relatively more expansive than the peripheries. This is also shown in Fig 1.

#### 3.2.2. Average affected age of household heads (AAA)

The cost of illness positively correlated with the age of household heads, which can also be called the average affected age of a household head. It was further learned from the analysis that cost of illness increased by PKR 86 (0.82 US$) as the average affected age increased by one year. Some household heads, especially older ones, had higher chances of getting infected from poor drinking water compared to young household heads.

#### 3.2.3. Incidence of Illness (IOI)

Interestingly, COI had some positive correlation with the incidence of illness in a household. As the incidence of illness increased from none to both, the cost of illness increased. Accordingly, COI increased by PKR 1498 (14.31 US$) if illness affected both genders of a household. There could be several vulnerabilities and reasons for such an increase in cost; for instance, when a female household member is ill, kitchen costs increase as she cannot cook and men rarely cook in Pakistan; therefore, food is ordered from outside. This may sometimes increase the cost. This is a real-world example; however, we do not have an analysis to support this phenomenon. It is purely based on experience and observation.

#### 3.2.4. Family size of households (FS)

COI and family size were positively related to each other and had a positive impact. The results show that an increase of one household member increased the COI by about PKR 103.2 (1 US$). This emphasizes the conclusion that a larger family with more members had more chances to get infected due to waterborne diseases as compared to smaller households in the studied area.

#### 3.2.5. Income of households (MI)

Another interesting fact is related to income and cost of illness. The results indicate that COI could be increased if there was an increase in monthly income of households. This is no wonder because according to the rule of thumb, whenever there is a positive shift in income, people will spend more on health. An ability to spend more usually increases with an increase in income, which is true for both peri-urban and urban households. An increase of one unit in income increases the COI by 3.89 US$ (about PKR 407.5). Such findings are truly in line which was found by Haq et al., (2007). Fig 1 further shows cost of illness and social status analysis in bubble graph manner to visualize such results.

#### 3.2.6. Household heads’ perception about the incidence of illness due to low quality drinking water (Suff)

**Fig 2:**
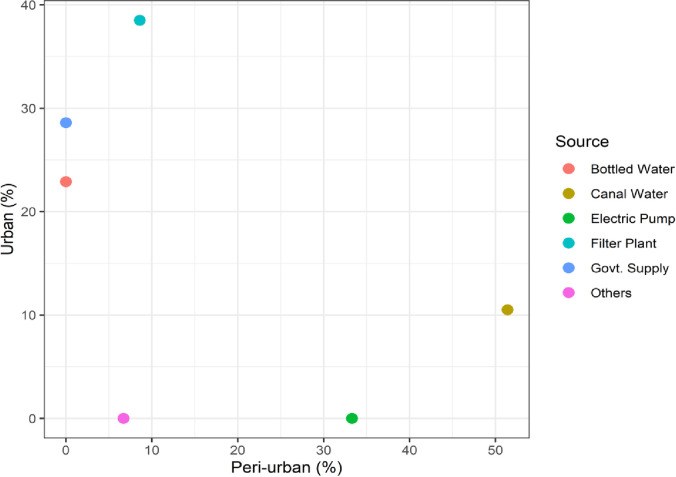
Distribution of drinking water sources of households among urban and peri-urban communities.

The survey investigated whether households placed importance on safe drinking water and water-related diseases. What perceptions did the households have? The answers were interestingly optimistic. A positive correlation was found between the perception of an increased incidence of COI and unsafe poor-quality water. Due to such a positive perception, the COI increased by 14.14 USD (PKR 1,480). These results show that households are fully aware that safe drinking water is a necessity and can reduce the cost of illness; they are also conscious of the quality of drinking water and related diseases. Fig 2 provides information on the different sources of drinking water available to households.

#### 3.2.7. Household heads’ satisfaction about the quality of drinking water (Satis)

Negative impacts on COI were found in the heads of households’ satisfaction levels regarding the quality of drinking water. This demonstrated that the satisfaction of household heads was negatively correlated to COI and water quality in the study area. As the satisfaction levels increased, the cost of illness reduced by almost up to 9.41 USD (PKR 985). This follows the results of household heads’ perceptions regarding the incidence of waterborne diseases. However, people feel at ease and relaxed if the satisfaction levels of households increase: in that way, people do not signify the incidence of illness, and prefer local or home remedies.

#### 3.2.8. Easy availability of quality drinking water (Avail)

**Fig 3:**
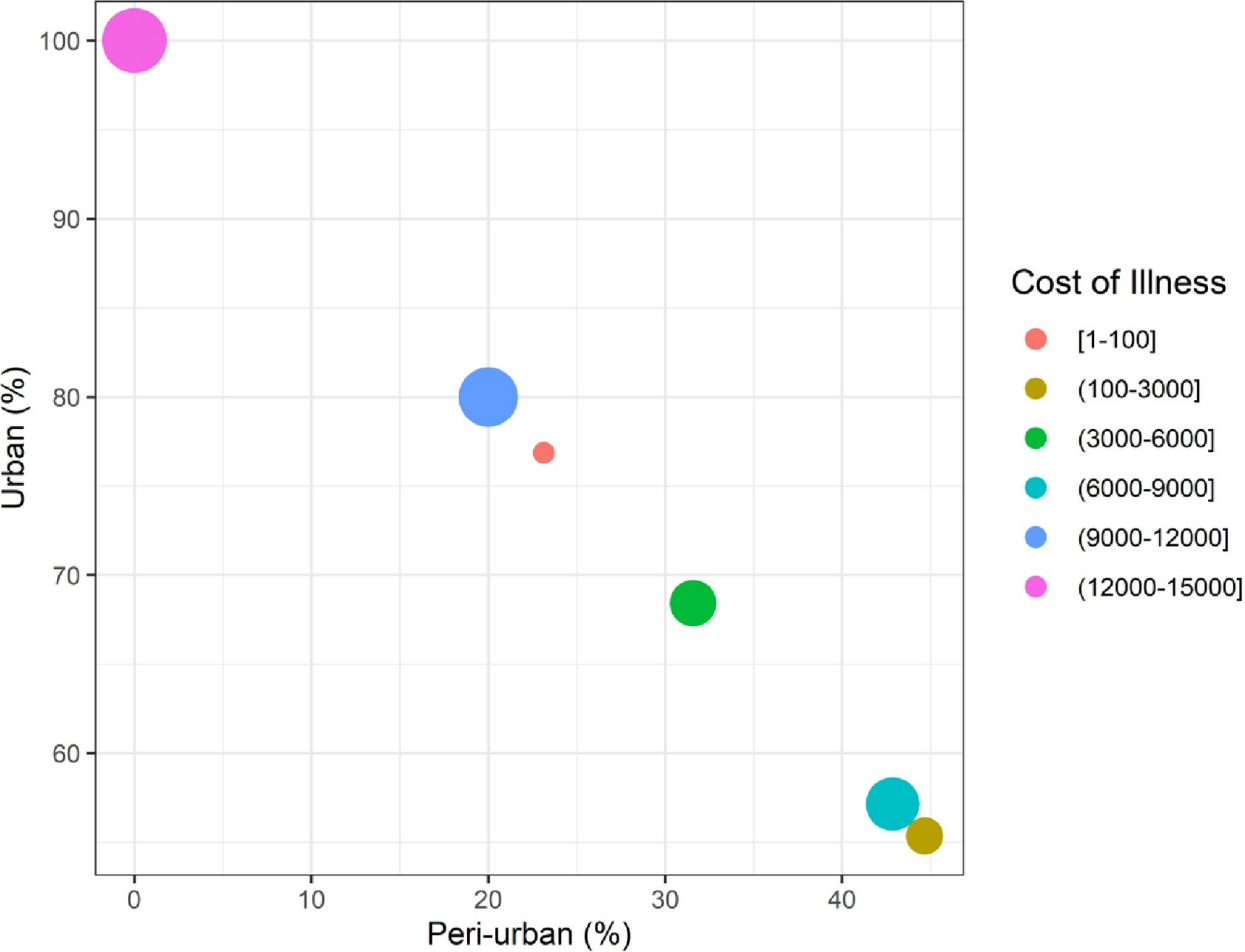
Distribution of cost of illness and willingness to pay of households among urban and peri-urban communities.

One of the serious issues found in the study areas was the availability of, and access to, clean drinking water. Both the cost of illness and the ease of acquiring quality drinking water had a negative impact. This issue is troublesome both in the peri-urban and urban areas of Faisalabad. If quality drinking water is readily available, the cost of illness reduces to 10.20 USD (almost PKR 1068). In our results, the ready availability of water showed a highly significant relationship with the cost of illness at (p < 0.003). Additionally, households were asked for payment which they are willing to pay to attain an improved quality of drinking water. As many as 50.5% of the households were willing to pay for quality drinking water, while 49.5% denied paying due to low affordability and low-income levels. Urban households were more willing to pay than peri-urban households. These results are also depicted in Fig 3.

### 3.3. Efficiency analysis

**Fig 4:**
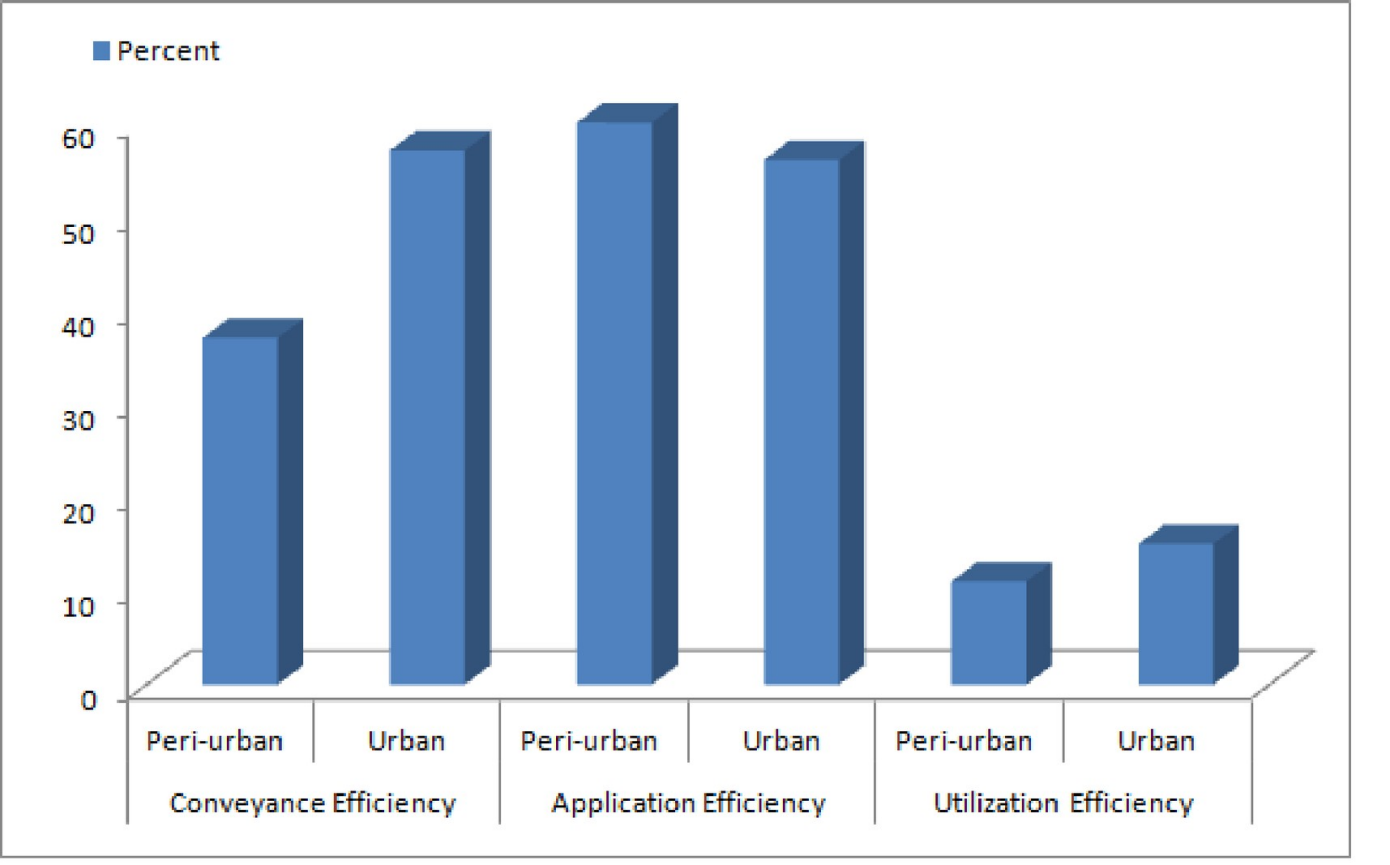
Efficiency of safe drinking water in urban and peri-urban areas.

The efficiency of drinking water was estimated in terms of conveyance, application, and usage efficiencies, following the estimation method of irrigation efficiency. The conveyance efficiency is concerned with the availability of drinking water and its consumption. Rogers et al., 1997, and Howell, 2013 [38–40], measured the irrigation conveyance efficiency, using ’water delivered to field’ and ’water diverted from source’ as variables. According to our results (presented in Fig 4), the residents of peri-urban areas were less efficient (37%) than the residents of urban areas (57%) in terms of conveyance efficiency. This is because peri-urban areas lack the facilities for quality drinking water.

Residents of urban areas were estimated to be less efficient (56%) as compared to their peri-urban area counterparts (60%). At the same time, households in urban areas were found to be less water application efficient (56%) with respect to application efficiency. This could be attributed to the fact that residents of peri-urban areas included laborers who required more water on account of their physical exertion. Another plausible reason could be that the availability of quality drinking water is not satisfactory to residents of peri-urban areas, which is they tend to be judicious in their water consumption.

Efficiency of water use is estimated in terms of cost of illness and daily water consumption. The residents of peri-urban areas were found to be less efficient (15%) than the residents of urban (11%). This is because the urban residents tend to bear less cost of illness. According to these findings, the residents of both peri-urban and urban areas are almost similar with regard to efficiency. Nonetheless, urban residents have an edge over their peri-urban counterparts when it comes to application efficiency. It is noteworthy that the residents of both these areas were inefficient in terms of utilization efficiency.

## 4. Conclusions

Clean and safe drinking water is one of the basic necessities of human beings. The consumption of contaminated water leads to waterborne diseases, which in turn cause social and economic losses. The results of the cost of illness/household estimation revealed that households of peri-urban areas spend more (US$ 10.79 (PKR 1725)) than the residents of urban areas i.e. US$ 6.84 (PKR 1094). The range of expenditures varies from zero expenditure to as high as US$ 69.42 (PKR 11,100) in peri-urban and US$ 93.19 (PKR 14,900) in urban areas. Furthermore, about 50% of the peri-urban residents and 90% of the urban residents are willing to pay PKR 100–1000 for quality/safe drinking water. It was found that the following had a positive correlation on the cost of illness: social status (residents of peri-urban), the average affected age of the household head, the average affected gender (female), family size, income, and household heads’ perception about the incidence of illness due to low quality drinking water. Residents of urban areas were slightly more efficient than the residents of peri-urban areas. A good majority of the respondents are willing to pay for quality drinking water. It shows that people are well aware of the importance of quality drinking water. Further studies should include the sanitation facilities in the households as well as the accessible hygiene materials and practices. It is suggested that family planning programs be made more effective to control family size. A good majority of the respondents are willing to pay for quality drinking water, it shows that people are well aware of the importance of quality drinking water. The filtration plants to enhance drinking water quality be installed in the central places of each town/division/union council. A public-private partnership could work to provide affordable quality drinking water.

## Supporting information

S1 File (DOCX)
