# Supplementary material for: Drinking-water efficiency, cost of illness, and peri-urban society: An economic household analysis
Source: PLoS One. 2021 Sep 29;16(9):e0257509. doi: 10.1371/journal.pone.0257509 (PMC8480852; doi:10.1371/journal.pone.0257509)
Supplement: S1 File — (DOCX) [file pone.0257509.s001.docx]

| **Supporting Information** | | | | | | | | | | | | |
| --- | --- | --- | --- | --- | --- | --- | --- | --- | --- | --- | --- | --- |
|  | Urban | | | | Peri-Urban | | | Total | | | | |
| **Monthly Income (PKR)** | | | | | | | | | | | | |
| < 10,000 | 0 | | | | 23 | | | 23 | | | | |
| 11,000-25,000 | 24 | | | | 64 | | | 88 | | | | |
| 26,000-40,000 | 33 | | | | 15 | | | 48 | | | | |
| 41,000-55,000 | 28 | | | | 3 | | | 31 | | | | |
| > 55,000 | 20 | | | | 0 | | | 20 | | | | |
|  | 105 | | | | 105 | | | 210 | | | | |
| **Type of Family** | | | | | | | | | | | | |
| Joint | 14 | | | | 16 | | | 30 | | | | |
| Nuclear | 91 | | | | 89 | | | 180 | | | | |
|  | 105 | | | | 105 | | | 2010 | | | | |
| **Household Size** | Min | | Mean | Max | Min | Mean | Max | Min | Mean | Max | | |
|  | 3 | | 6 | 16 | 4 | 8 | 22 | 3 | 7 | | | 22 |
| **Education (No. of Years)** | Min | Mean | | Max | Min | Mean | Max | Min | Mean | | Max | |
|  | 0 | 13 | | 22 | 0 | 3 | 10 | 0 | 8 | | 22 | |
| Illiterate (0) | 1 | | | | 55 | | | 56 | | | | |
| Primary (5) | 4 | | | | 28 | | | 32 | | | | |
| Middle (8) | 2 | | | | 10 | | | 12 | | | | |
| Metric (10) | 15 | | | | 12 | | | 27 | | | | |
| Intermediate (12) | 17 | | | | 0 | | | 17 | | | | |
| Bachelor’s (14) | 32 | | | | 0 | | | 32 | | | | |
| Masters (16) | 26 | | | | 0 | | | 26 | | | | |
| M.Phil. (18) | 6 | | | | 0 | | | 6 | | | | |
| Ph.D. (22) | 2 | | | | 0 | | | 2 | | | | |
|  | 105 | | | | 105 | | | 210 | | | | |
| **Gender (HHH)** | | | | | | | | | | | | |
| Male | 97 | | | | 92 | | | 189 | | | | |
| Female | 8 | | | | 13 | | | 21 | | | | |
|  | 105 | | | | 105 | | | 210 | | | | |
| **Incidence of Illness** | | | | | | | | | | | | |
| None | 83 | | | | 55 | | | 138 | | | | |
| Females | 6 | | | | 25 | | | 31 | | | | |
| Males | 13 | | | | 17 | | | 30 | | | | |
| Both | 3 | | | | 8 | | | 11 | | | | |
|  | 105 | | | | 105 | | | 210 | | | | |
| **Source of Drinking Water** | | | | | | | | | | | | |
| Ground water | 0 | | | | 35 | | | 35 | | | | |
| Others (Filter plant, Canal water, Govt. supply, bottled water i.e. nestle etc.) | 105 | | | | 70 | | | 175 | | | | |
|  | 105 | | | | 105 | | | 210 | | | | |
| **Awareness about the importance of safe drinking water (Aw)** | | | | | | | | | | | | |
| No | 10 | | | | 79 | | | 89 | | | | |
| Yes | 95 | | | | 26 | | | 121 | | | | |
|  | 105 | | | | 105 | | | 210 | | | | |
| **Household heads' perception about the incidence of illness due to low quality drinking water (Suff)** | | | | | | | | | | | | |
| No | 83 | | | | 52 | | | 135 | | | | |
| Yes | 22 | | | | 53 | | | 75 | | | | |
|  | 105 | | | | 105 | | | 210 | | | | |
